# Is an Electronic Nose Able to Predict Clinical Response following Neoadjuvant Treatment of Rectal Cancer? A Prospective Pilot Study

**DOI:** 10.3390/jcm13195889

**Published:** 2024-10-02

**Authors:** Ivonne J. H. Schoenaker, Alexander Pennings, Henderik L. van Westreenen, Evelyn J. Finnema, Richard M. Brohet, Julia Hanevelt, Wouter H. de Vos Tot Nederveen Cappel, Jarno Melenhorst

**Affiliations:** 1 Oncology Center Isala, Isala Dokter van Heesweg 2, 8025 AB Zwolle, The Netherlands; i.j.h.schoenaker@isala.nl; 2 Department of Health Science, University Medical Center Groningen, Section of Nursing Research, Hanzeplein 1, 9713 GZ Groningen, The Netherlands; e.j.finnema@umcg.nl; 3 Department of Surgery, Maastricht University Medical Center, P. Debyelaan 25, GROW School for Oncology and Reproduction, Maastricht University, 6229 HX Maastricht, The Netherlandsjarno.melenhorst@mumc.nl (J.M.); 4 Department of Surgery, Isala, Dokter van Heesweg 2, 8025 AB Zwolle, The Netherlands; h.l.van.westreenen@isala.nl; 5 Department of Epidemiology & Statistics, Isala, Dokter van Heesweg 2, 8025 AB Zwolle, The Netherlands; r.m.brohet@isala.nl; 6 Department of Gastroenterology and Hepatology, Isala, Dokter van Heesweg 2, 8025 AB Zwolle, The Netherlands; j.hanevelt@isala.nl

**Keywords:** rectal cancer, electronic nose, volatile organic compounds, neoadjuvant treatment, response evaluation

## Abstract

**Introduction**: A watch-and-wait strategy for patients with rectal cancer who achieve a clinical complete response after neoadjuvant (chemo) radiotherapy is a valuable alternative to rectal resection. In this pilot study, we explored the use of an electronic nose to predict response to neoadjuvant therapy by analyzing breath-derived volatile organic compounds. **Materials and Methods:** A pilot study was performed between 2020 and 2022 on patients diagnosed with intermediate- or high-risk rectal cancer who were scheduled for neoadjuvant therapy. Breath samples were collected before and after (chemo) radiotherapy. A machine-learning model was developed to predict clinical response using curatively treated rectal cancer patients as controls. **Results**: For developing the machine-learning model, a total of 99 patients were included: 45 patients with rectal cancer and 54 controls. In the training set, the model successfully discriminated between patients with and without rectal cancer, with a sensitivity and specificity of 0.80 and 0.65, respectively, and an accuracy of 0.72. In the test set, the model predicted partial or (near) complete response with a sensitivity and specificity of 0.64 and 0.47, respectively, and an accuracy of 0.58. The AUC of the ROC curve was 0.63. **Conclusions**: The prediction model developed in this pilot study lacks the ability to accurately differentiate between partial and (near) complete responders with an electronic nose. Machine-learning studies demand a substantial number of patients and operate in a rapidly evolving field. Therefore, the prevalence of disease and duration of a study are crucial considerations for future research.

## 1. Introduction

Each year, there are approximately 700,000 new cases of rectal cancer worldwide [1]. To reduce the risk of local recurrence in the future and to enable an organ-preserving treatment, patients with intermediate- or high-risk rectal cancer are treated with neo-adjuvant therapy to downstage the tumour. Neo-adjuvant therapy consists of short-course radiotherapy (scRT) or chemoradiotherapy (CRT). Response evaluation is performed 8 weeks after the last radiotherapy dose, including digital rectal examination, MRI, and a sigmoidoscopy. In 20 to 30% of patients, a clinical complete response is achieved. With these patients, a close surveillance programme, the so-called “Watch&Wait program” (W&W) is discussed as an option instead of a radical resection. In patients with a near-complete response, a second response evaluation is scheduled after a further 6–12 weeks of waiting. After this period, more patients (90%) will become complete responders, while others will more clearly show residual tumour at this time [2,3]. As a clinical response evaluation with current methods is not completely accurate, properly selecting patients for a W&W strategy remains challenging. Digital rectal examination and sigmoidoscopy are more accurate than MRI, and combining these methods achieves the highest accuracy, an AUC of 0.91 [4,5]. In total, 15 to 30% of the clinical complete responses are not recognized with current response evaluation methods, and these patients may undergo unnecessary surgery. The lack of diagnostic accuracy increases patient uncertainty and healthcare costs [4,5]. This justifies the search for better diagnostic tools to assess response after neo-adjuvant treatment.

Analysis of volatile organic compounds (VOCs) in exhaled air is gaining interest in the field of colorectal cancer detection. Each individual has been shown to have a personal ’breathprint’, comparable to a fingerprint, which is a reflection of health. These breathprints consists of volatile organic compounds (VOCs), which are gaseous products of metabolism [6]. VOCs can be used as biomarkers for diseases, including several cancers [7,8,9]. Specific VOC changes are recognized in patients with colorectal cancer (CRC) [10]. The presence of CRC changes the overall endogenous metabolism, resulting in the release of a specific composition of VOCs in the exhaled air [6,11,12]. Besides an underlying condition, VOCs composition could also change through treatment as radiotherapy or lifestyle [13,14].

Electronic nose devices (eNose) can be used to analyze VOC profiles in exhaled air by pattern-recognition techniques with artificial intelligence (AI). An eNose is a hand-held, non-invasive, low-cost, and real-time diagnostic tool. Van Keulen et al. published data on the use of eNose in the discrimination between patient with CRC and advanced adenomas from healthy controls, with an accuracy of 0.84 and 0.73, respectively [7]. Earlier studies have also demonstrated that eNose can detect recurrence of CRC with a diagnostic accuracy of 0.81 [10]. Additionally, we demonstrated alteration in VOC patterns following curative surgery for CRC, with an accuracy of 0.75 [15,16].

This pilot study aimed to assess the diagnostic performance of eNose technology in predicting response following neo-adjuvant treatment by discriminating between partial or (near) complete response based on the analysis of VOCs in the breath of patients with rectal cancer.

## 2. Material and Methods

### 2.1. Study Design and Patient Selection

A prospective pilot study was conducted in two Dutch hospitals: Maastricht University Medical Center (MUMC) and Isala Zwolle, between 2020 and 2022. To predict the clinical response to neoadjuvant therapy, we first developed a machine-learning model capable of differentiating between patients with and without rectal cancer, based on their VOC pattern detected with the eNose. The clinical response, as determined in the multi-disciplinary board was used as reference and was classified as a partial, near-complete, or complete response [2,3].

Two groups of patients were involved, denoted as Group A and Group B. All tests were performed with the same device at each location to avoid confounding factors related to the device. Group A consisted of newly diagnosed patients with intermediate- (cT1-3 MRF—N1) or high-risk rectal cancer (cT4, MRF +, cN2, extra mesorectal pathological lymph node(s), and/or EMVI), scheduled for neoadjuvant therapy. Neoadjuvant therapy options included scRT (5 fractions of 5 Gy) or CRT (25 fractions of 2 Gy combined with capecitabine bid 2 × 850 mg/m^2^). Exclusion criteria were synchronous metastases, a history of another malignancy in the past five years (except for basal-cell carcinoma), inability to perform the breath test, or insufficient understanding of the Dutch language. The patients in group A underwent two breath tests; one was performed at diagnosis, and the second was after the neoadjuvant therapy at response evaluation. Group B was used as a control group and consisted of patients who were curatively treated with neoadjuvant therapy for rectal cancer with no signs of recurrence of disease. Patients were in follow-up during the current study, either after resection or having close surveillance in the W&W programme. Breath samples were available from these patients, as they participated in other eNose studies in the two research centers. Exclusion criteria for inclusion in group B were earlier participation in group A, recurrence of disease during follow-up, diagnosis of another malignancy during follow-up, and using a different electronic device for the breath test.

**Figure 1:**
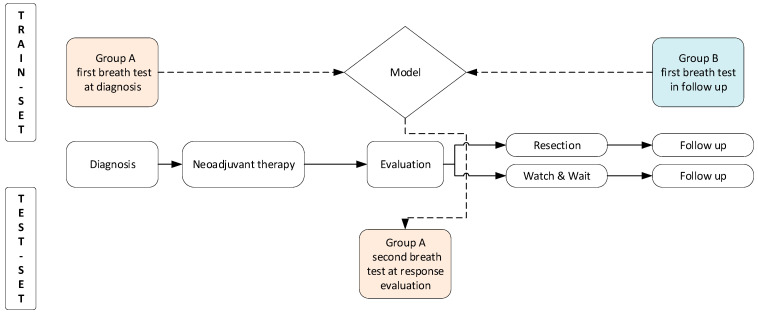
Study design: A machine-learning model was developed by discriminating between group A (=newly diagnosed patients with rectal cancer), first breath sample, and group B (=patient treated for rectal cancer), breath test during follow-up. This is called the training set. The model was evaluated on the test set. These breath tests were performed at response evaluations approximately 8 weeks after last radiotherapy.

A machine-learning model was developed that could discriminate between group A’s initial breath sample at diagnosis and group B’s breath test during follow-up, referred to as the training set. Using this developed model, the response to neoadjuvant treatment was predicted based on the second breath test of group A, referred to as the test set (Figure 1).

### 2.2. Study Procedures

Before breath samples were taken, exogenous factors that might influence the VOC composition, like smoking, medication, alcohol, or fasting time, and endogenous patient characteristics, like the patient’s Body Mass Index (BMI) or specific comorbidities, were collected [13]. Patients breathed into the device for 5 min through a disposable mouthpiece provided with carbon filters that prevent contamination of the inhaled air with environmental VOCs. All patients wore a nose clip during the 5 min of breathing and were instructed to close their lips firmly around the disposable mouthpiece to avoid pollution with unfiltered air. To standardize the execution of the breath test, all healthcare practitioners executing the breath test were instructed during a short instruction class by The eNose Company.

### 2.3. Aeonose™ Technology and Model Development

Breath tests were conducted with two CE-certified Aeonose^TM^ devices from The eNose Company (Zutphen, The Netherlands). The Aeonose^TM^ technology has successfully been used and described for lung cancer diagnosis [17,18,19]. The technological aspects of the eNose are discussed in detail by Hanevelt et al. [16]. The performance of different machine-learning models were evaluated by the proprietary software programme ‘Aethena’ version 2.64 [16,17].

The model was developed using the training set, consisting of group A, patients with rectal cancer, and group B, patients without rectal cancer after treatment. The model performance was validated using the test set, consisted of patients of group A, at the second breath test. The test set was used to predict the response of the neo-adjuvant treatment. The response was defined as a partial or (near) complete response. Due to the small sample size, training the model may encounter high variance and it might be more prone to overfitting. The performance of each model was evaluated, and the most optimal model was chosen, which was a model based on a Random Forest algorithm. During the analysis, a cut-off value was determined, showing the best separation between the two groups regarding optimal sensitivity and specificity.

### 2.4. Statistical Analysis

Patient characteristics were summarized by count and proportion for categorical data, by mean and standard deviation for normally distributed continuous data or median and interquartile range for non-normal distributed data. T-tests, Mann–Whitney U test, Chi-squared (X^2^), or Fishers exact test were applied as appropriate to assess differences between the groups Normality was verified using a Shapiro–Wilk test. A (two-sided) *p*-value < 0.05 was considered significant.

Although an exact sample-size calculation is unfeasible for machine-learning studies, previous data implied that at least 25 samples per group are required to develop a solid model in the training phase [16]. An ideal diagnostic test should be both sensitive and specific. Performance metrics included sensitivity, specificity, accuracy, positive predictive value (PPV), negative predictive value (NPV), and the area under the curve (AUC) of the ‘receiver-operating characteristics curve (ROC-curve)’. An AUC ≥ 0.7 is considered as an acceptable performance. All analyses were performed using Statistical Package of Social Sciences version 24.0 (SPSS, IBM, Armonk, NY, USA).

### 2.5. Ethics

The Medical Ethics Review Committee (METC) of Isala, Zwolle, has declared that the above study protocol is not subject to the Medical Research Involving Human Subjects Act (WMO) (Isala METC 200120). Informed consent was obtained from all participants before sampling.

## 3. Results

### 3.1. Study Population

**Figure 2:**
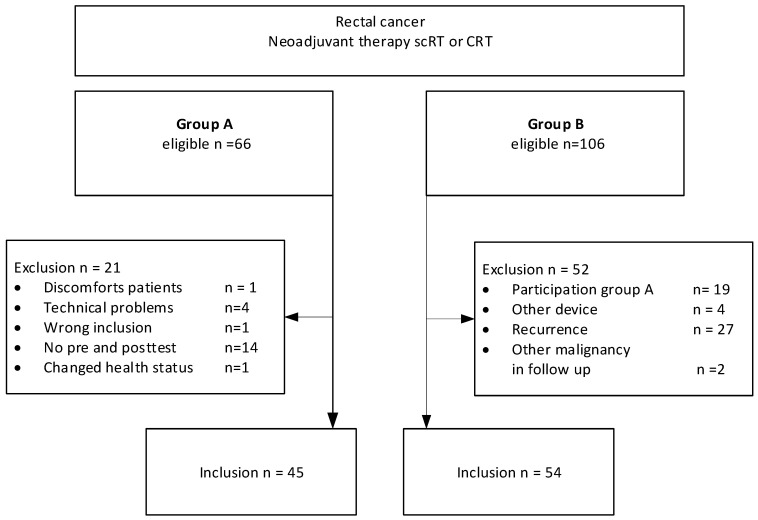
Patient enrollment.

In group A, 66 patients with newly diagnosed rectal cancer were enrolled between October 2020 and November 2022. Twenty-one patients were excluded for various reasons, including failed or missing breath tests, technical difficulties, incorrect inclusion, or changes in health status. Among these patients, fourteen were excluded because they only completed one breath test, due to the temporary suspension of research during the COVID-19 pandemic. This resulted in a total of 45 patients in group A. In total, 106 patients were initially eligible for inclusion in Group B. Following exclusions, 54 patients remained included (Figure 2).

### 3.2. Patient Characteristics

The baseline patient characteristics of both groups are shown in Table 1. The mean age of the 99 included patients was 65 years, and gender was equally divided, male 52% vs. female 49%. There were no significant differences in characteristics between both groups, except for a larger proportion of high-risk stage (*p* = 0.023), more CRT (*p* = 0.010), and more frequent fasting before breath test (*p* = 0.043) in Group B.

### 3.3. Model Performance of the Training Set

To obtain the most optimal discrimination performance in the training set, the threshold was set at 0.00, meaning that all breath tests with a predicted value of > 0.00 were still classified as presence of rectal cancer. The model in the training set was able to discriminate between patients with or without rectal cancer with a sensitivity and specificity of 0.80 (95% CI 0.65–0.9) and 0.65 (95% CI 0.51–0.77), respectively, and an accuracy of 0.72 (95% CI 0.63–0.80). The AUC of the ROC curve was 0.86 (95% CI 0.79–0.93) (Table 2).

### 3.4. Response Evaluation

During the response evaluation by the multidisciplinary board conducted eight weeks after neoadjuvant treatment, 28 out of 45 patients of group A were classified as partial responders and 17 as (near) complete responders. Among the partial responders, 27 underwent surgery and were found to have residual tumour in the resection specimen, while one patient died between the second breath test and a surgery not related to rectal cancer. This indicates that in 96% of cases, the classification of a partial response by the multidisciplinary board was pathologically confirmed.

Of the 17 (near) complete responders, 15 are still included in the W&W follow-up and have shown no regrowth at a minimum follow-up of 9 months, indicating that 88% of the patients were correctly classified as (near) complete responder after response evaluation. One of the 15 patients in W&W patient underwent an endoscopic luminal resection of a tubulovillous adenoma with high grade dysplasia. Among the two patients who were no longer in the W&W follow-up, one underwent surgery after multiple evaluations indicated a possible residual tumour. After the operation, this patient was classified as ypT0N0. The other patient experienced regrowth after three months in the W&W follow-up and was reclassified as ypT2N0Mx after resection.

### 3.5. Model Performance of the Test Set

**Figure 3:**
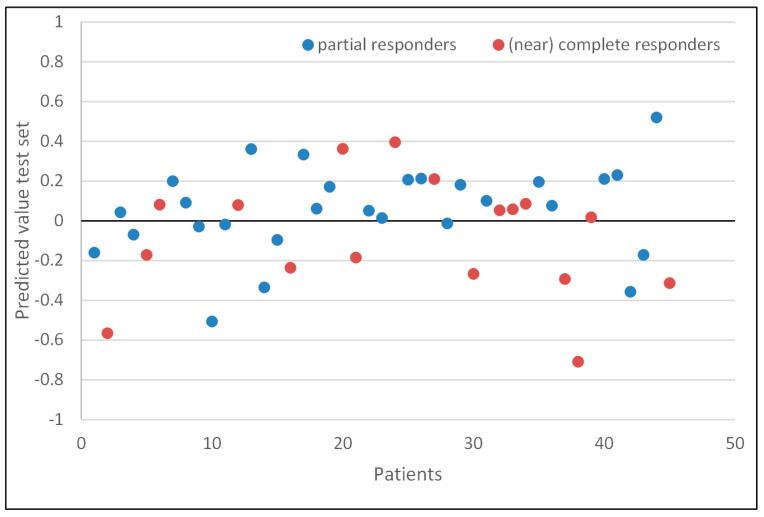
Scatterplot of individual predictive values of each patient in the test set. The threshold is set on 0.00. Values > 0.00 are scored as partial response. Blue dots are patients with a partial response and red are patients with a (near) complete response.

The mean time between the last dose of radiotherapy and the second response evaluation breath test of group A was 56 days (±SD 12). Figure 3 shows the individual predictive values of each patient in the test set. Among the 28 partial responders, 18 were identified as true positive, while 10 were falsely predicted as negative by the model. Of the 17 (near) complete responders, 8 patients were accurately identified as true negative, while 9 were incorrectly predicted as positive.

The test model was able to predict between partial response and (near) complete response, with a sensitivity and specificity of 0.64 (95% CI 0.46–0.82) and 0.47 (95% CI 0.23–0.7), respectively, and an accuracy of 0.58 (Table 2). The AUC of the ROC curve was 0.63 (95% CI 0.45–0.81). Sensitivity, specificity, negative predictive value (NPV), and accuracy were lower in the test set. Only the positive predictive value (PPV) was similar in the train- and test sets.

No significant differences in demographic, clinical characteristics, and measurement conditions (for example, fasting 3 h before the breath test, use of alcohol, diet, use of supplements, medicine use, and location) were found between the correctly and incorrectly predicted breath samples of the positive group (Table 3).

## 4. Discussion

While the machine-learning model looked promising in the training set with an AUC of 0.86, its performance in the test set was much lower, with an AUC of 0.63. To our knowledge, this is the first study using eNose technology to predict response following neoadjuvant treatment for rectal cancer. This pilot study aimed to examine the ability of Aeonose™ to discriminate between patients with a partial or (near) complete response at response evaluation.

Several explanations may be the cause for the low AUC in the test set. First, the robustness of the developed machine-learning model may have been insufficient. The ability of a model to perform well on new and unseen data should be at least similar to the performance in the training set. However, in this pilot study, all performance metrics were lower in the test set compared to the training set. Our primary objective was not to establish a robust model before predicting blind samples, and the limited sample size of this pilot study might contribute to the low performance of the model in the test set. Concerning this, possible future studies may profit from the methodology as suggested by Haalboom et al. [20].

Another explanation might be a difference between changes in metabolism between the train and test set. Although the extent of this influence is not well-documented, VOC patterns are known to be influenced by changes in metabolism [13,14]. In the present study, various factors, such as measurement conditions, lifestyle factors, radiotherapy, and the interval between neoadjuvant treatment and breath tests, could contribute to alterations and differences between train and test set. For instance, the downstaging of the tumour through neoadjuvant therapy induces processes such as necrosis, inflammation, ulceration, and oxidative stress, all of which can influence the measured VOC profile. Patients with a (near) complete response often exhibit ulcers or fibrosis at the tumour site [3]. This may result in a VOC profile that differs from the one the model was developed on.

In this study, patients received different doses of radiotherapy, either a total of 25 Gy for the scRT or 50 Gy for the CRT. There was no significant difference in radiotherapy doses between the correctly and incorrectly predicted patients. Philips et al. identified 15 VOCs that appear after exposure to localized doses of 1.8 Gray and higher, suggesting that radiotherapy influences the composition of different VOCs in exhaled air [14]. It should be noted that the VOCs in the study of Philips et al. were measured during radiotherapy treatment. In contrast, our study measured the VOCs during response evaluation with a mean of 56 days after the last dose of radiotherapy. It is possible that radiotherapy may have another influence in the longer term.

In our study, half of the (near) complete responders required a second or even third response evaluation, as some patients took longer to achieve a complete response. As a result, minimal tumour load might be still present at the time of the breath test after neoadjuvant treatment, affecting the results. We did not collect data on potential tumour, ulceration, or scars assessed by pelvis MRI and sigmoidoscopy. Future research could explore changes in VOCs over time, including not only breath tests at diagnosis and response evaluation but also during second and third response evaluations and after treatment.

It is known that VOCs composition could also change through lifestyle. Bosch et al. investigated lifestyle factors affecting volatile organic compounds (VOCs), including age, gender, smoking, diet, comorbidity, and medication usage [13]. While all lifestyle factors impacted the VOCs, the most substantial influences were observed in individuals with a low Body Mass Index (BMI), those following a vegetarian diet, and active smokers. Unfortunately, our study lacked a sufficient number of patients with a vegetarian diet or a low BMI to explore these factors’ influence. Although the proportion of active smokers was higher among correctly predicted patients, the difference did not reach statistical significance. It is important to note differences between our study and that of *Bosch* et al. Their investigation focused on fecal VOCs, employing a different eNose device, the Cyranose©320. Additionally, their study exclusively involved patients without colon abnormalities, whereas our cohort exhibited colon abnormalities attributed to rectal cancer and treatment consequences as necrosis, inflammation, and ulceration.

The strength of this study was the usage of both a homogenous group of patients with and without rectal cancer to develop the machine-learning model. Furthermore, this was a multicenter study, and only one device was used at each location where the breath tests were taken.

A limitation of our study is that we did not perform an overfitting correction or an internal validation of our training set. Even though this pilot study contained a small number of included patients, the initial aim to have a minimum of 25 patients with and 25 patients without rectal cancer for the training set was met. In addition, *The eNose Company* provided directions during the study to increase the number of patients needed per device at each location. Consequently, instead of 25 patients with and without rectal cancer at both hospital locations combined, 25 patients with and without rectal cancer at each hospital were necessary. Unfortunately, these target numbers were not met. One contributing factor was the relatively low prevalence of (near) complete responders. Obtaining enough (near) complete responders took longer than we anticipated. Additionally, another study investigating an alternative neoadjuvant treatment strategy for patients with high-risk rectal cancer commenced during our study period, resulting in fewer eligible patients for our study.

## 5. Conclusions

The prediction model developed in this pilot study lacks the accuracy to discriminate between partial responders and (near) complete responders. Therefore, no added value to the current response evaluation modalities were observed. Enhancing the model’s performance could potentially change this outcome in the future. Considering the rapid advancement in machine learning studies and their need for extensive datasets, careful deliberation regarding the study’s prevalence and duration is crucial for future research.

## Figures and Tables

**Table 1:** Baseline patient characteristics.

	Total	Group A	Group B	*p*-Value
	*n* = 99	*n* = 45	*n* = 54	
**Age, mean ± SD**	65 ± 9.7	66 ± 9.0	65 ± 10.6	0.470
**Gender, *n* (%)**				0.199
Male	51 (52)	20 (44)	31 (57)	
Female	48 (49)	25 (56)	23 (43)	
**BMI kg/m^2^, *n* (%)**				0.288
<25	31 (31)	14 (31)	17 (32)	
25–29.9	46 (47)	24 (53)	22 (41)	
≥30	22 (22)	7 (16)	15 (28)	
**Current smoker, *n* (%)**	10 (10)	6 (13)	4 (7)	0.505
**ASA, *n* (%)**				0.530
I	29 (29)	11 (24)	18 (33)	
II	67 (68)	32 (71)	35 (65)	
III	3 (3)	2 (4)	1 (2)	
**MMR-status, *n* (%)**				0.900
MMR-Proficient	75 (76)	35 (78)	40 (74)	
MMR-Deficient	1 (1)	0	1 (2)	
Unknown	23 (23)	10 (22)	13 (24)	
**Risk stage, *n* (%)**				0.023
Low risk	7 (7)	6 (13)	1 (2)	
Intermediate risk	27 (27)	15 (33)	12 (22)	
High risk	65 (66)	24 (53)	41 (76)	
**Neo-adjuvant treatment, *n* (%)**				0.010
scRT	29 (29)	19 (42)	10 (19)	
CRT	70 (71)	26 (58)	44 (82)	
**Response evaluation, *n* (%)**				0.764
Partial	60 (61)	28 (62)	32 (59)	
(Near) complete	39 (39)	17 (38)	22 (41)	
**Location, *n* (%)**				0.055
Zwolle	69 (70)	27 (60)	42 (78)	
Maastricht	30 (30)	18 (40)	12 (22)	
**Comorbidity, *n* (%)**				
Hypertension	25 (25.3)	11 (24.4)	14 (25.9)	0.866
Diabetes Mellitus	16 (16.2)	9 (20)	7 (13)	0.344
**Medication, *n* (%)**				
PPI use	13 (13)	8 (18)	5 (9)	0.195
Metformin use	13 (13)	7 (16)	6 (11)	0.486
**Use of supplements, *n* (%)**	31 (31.3)	18 (40)	13 (24)	0.089
**Last meal < 3 h, *n* (%)**	42 (43.3)	24 (54.5)	18 (34)	0.043
**Alcohol use, *n* (%)**	66 (68)	26 (60.5)	40 (74.1)	0.153
**Alcohol < 24 h, *n* (%)**	33 (34.4)	11 (25.6)	22 (41.5)	0.102

BMI: Body Mass Index; ASA: American Society of Anesthesiologists; MMR: mismatch repair; PPI: proton pump inhibitors.

**Table 2:** Performance prediction model 95% CI.

	Training Set *n* = 99	Test Set *n* = 45
**Sensitivity**	0.80 (CI 0.65–0.90)	0.64 (CI 0.46–0.82)
**Specificity**	0.65 (CI 0.51–0.77)	0.47 (CI 0.23–0.70)
**PPV**	0.65 (CI 0.51–0.77)	0.67 (CI 0.49–0.84)
**NPV**	0.80 (CI 0.64–0.90)	0.44 (CI 0.21–0.67)
**Accuracy**	0.72 (CI 0.63–0.80)	0.58 (CI 0.43–0.72)
**AUC**	0.86 (CI 0.79–0.93)	0.63 (CI 0.45–0.81)

PPV: positive predictive value; NPV: negative predictive value; AUC: area under the curve.

**Table 3:** Baseline characteristics of the correctly predicted versus incorrectly predicted patients at the response evaluation.

	Total	Correctly Predicted	Incorrectly Predicted	*p*-Value
	*n* = 45	*n* = 26	*n* = 19	
**Age, mean ± SD**	66 ± 9.0	65 ± 10.4	67 ± 6.9	0.370
**Gender, *n* (%)**				0.345
Male	20 (44)	10 (39)	10 (53)	
Female	25 (56)	16 (62)	9 (47)	
**BMI kg/m^2^, *n* (%)**				0.380
<25	14 (31)	10 (39)	4 (21)	
25–30	24 (53)	12 (46)	12 (63)	
≥30	7 (16)	4 (15)	3 (16)	
**Current smoker**	6 (13)	5 (19)	1 (5)	0.222
**ASA, *n* (%)**				0.056
I	11 (24)	4 (15)	7 (37)	
II	32 (71)	20 (77)	12 (63)	
III	2 (4)	2 (8)	0	
**Risk Stage, *n* (%)**				0.557
Low risk	6 (13)	3 (12)	3 (16)	
Intermediate risk	15 (33)	11 (42)	4 (21)	
High risk	24 (53)	12 (46)	12 (63)	
**Neo-adjuvant treatment, *n* (%)**				0.532
scRT	19 (42)	12 (46)	7 (37)	
CRT	26 (58)	14 (54)	12 (63)	
**Response evaluation, *n* (%)**				0.257
Partial	28 (62)	18 (69)	10 (53)	
(near) complete	17 (38)	8 (31)	9 (47)	
**Location, *n* (%)**				0.712
Zwolle	27 (60)	15 (58)	12 (63)	
Maastricht	18 (40)	11 (42)	7 (37)	
**Comorbidity, *n* (%)**				
Hypertension	11 (24)	7 (27)	4 (21)	0.736
Diabetes Mellitus	9 (20)	8 (31)	1 (5)	0.058
**Medication, *n* (%)**				
PPI use	8 (18)	6 (24)	2 (11)	0.433
Metformin use	7 (16)	6 (24)	1 (5)	0.119
**Last meal < 3 h, *n* (%)**	24 (55)	14 (56)	10 (53)	0.824
**Use of supplements, *n* (%)**	18 (40)	12 (46)	6 (32)	0.324
**Alcohol use, *n* (%)**	26 (61)	13 (54)	13 (68)	0.342
**Alcohol < 24 h, *n* (%)**	11 (26)	5 (21)	6 (32)	0.495

BMI: Body Mass Index; ASA: American Society of Anesthesiologists; MMR: mismatch repair; PPI: proton pump inhibitors.

## Data Availability

The data presented in this study are available on request from the corresponding author.

## Abbreviations

scRT	Short-course radiotherapy
CRT	Chemoradiotherapy
W&W	Watch-and-Wait
VOC	Volatile organic compounds
CRC	Colorectal cancer
eNose	Electronic nose
